# Dose-response models for selected respiratory infectious agents: *Bordetella pertussis*, group a *Streptococcus*, rhinovirus and respiratory syncytial virus

**DOI:** 10.1186/s12879-015-0832-0

**Published:** 2015-02-24

**Authors:** Rachael M Jones, Yu-Min Su

**Affiliations:** Division of Environmental and Occupational Health Sciences, School of Public Health University of Illinois, Chicago, USA

**Keywords:** Risk assessment, Dose-response, Respiratory infections, Pharyngitis, Common cold

## Abstract

**Background:**

Dose-response assessment is one step in quantitative microbial risk assessment (QMRA). Four infectious microbes capable of causing respiratory diseases important to public health, and for which dose-response functions have not been available are: *Bordetella pertussis* (whooping cough), group A *Streptococcus* (pharyngitis), rhinovirus (common cold) and respiratory syncytial virus (common cold). The objective of this study was to fit dose-response functions for these microbes to published experimental data.

**Methods:**

Experimental infectivity data in human subjects and/or animal models were identified from the peer-reviewed literature. The exponential and beta-Poisson dose-response functions were fitted using the method of maximum likelihood, and models compared by Akaike’s Information Criterion.

**Results:**

Dose-response functions were identified for each appropriate data set for the four infectious microbes. Statistical and graphical measures of fit are presented.

**Conclusions:**

With the fitted dose-response functions it will be possible to perform QMRA for these microbes. The dose-response functions, however, have a number of limitations associated with the route of exposure, use of animal hosts, and quality of fit. As a result, thoughtfulness must be used in selecting one dose-response function for a QMRA, and the function should be recognized as a significant source of uncertainty. Nonetheless, QMRA offers a transparent, systematic framework within which to understand the mechanisms of disease transmission, and evaluate interventions.

**Electronic supplementary material:**

The online version of this article (doi:10.1186/s12879-015-0832-0) contains supplementary material, which is available to authorized users.

## Background

Quantitative microbial risk assessment (QMRA) is a growing and diversifying area of research for public health. QMRA seeks to evaluate the risk of adverse health effects, particularly infection, resulting for human exposures to infectious microbes. Applications to water-borne and food-borne infectious microbes are too numerous to cite, and have enhanced public health decision making through exploration of: scenarios that cannot be directly observed [1], the effectiveness of interventions [2], and causes of infectious disease outbreaks [3]. In the context of microbes capable of causing respiratory infections, quantitative microbial risk assessment research has focused on influenza [4-8] and tuberculosis, often in the context of transportation [5,9-11]. One reason for limited application of QMRA to microbes capable of causing respiratory infections may be the limited availability of dose-response functions, which are complicated by uncertainty about mechanisms of disease transmission.

Dose-response functions are mathematical expressions that characterize the probability of an adverse health outcome (e.g., infection) subsequent to an exposure. Dose-response functions are fitted to adverse health outcomes observed among animals or human volunteers exposed to graded doses of infectious microbes. Though the conceptual models underlying dose-response functions differ between microbes and chemical agents [12], the nature of data and use of dose-response functions in risk assessment are similar. Dose-response assessment – the identification and/or development of a dose-response function – is one step in the classical risk assessment paradigm of: hazard identification, dose-response assessment, exposure assessment, and risk characterization. This paper focuses on the development of dose-response functions, and discusses issues around the selection and interpretation of dose-response functions in animal models and exposure routes for human disease transmission. Completion of a QMRA requires substantial additional information and analysis.

Absent a dose-response function, it is possible to apply the concept of the quantum of infection to estimate the risk of infection. This approach is commonly used for *Mycobacterium tuberculosis*, but has been applied for other infectious microbes transmitted through the inhalation of airborne microbes [5,13-15]. The quantum of infection is an unspecified number of microbes that has the effect of causing infection [16]. If the rate of emission is also measured in quanta of infection, such as when the emission rate is estimated from observed disease or tubercule formation [17], then it is possible to estimate risk without an explicit dose-response function. For example, the Wells-Riley equation may be applied. The Wells-Riley equation estimates the probability of infection among susceptible people as an exponential function of the rate at which quanta of infection are emitted into a well-mixed, ventilated room [18]. Given a single infectious source emitting *q* quanta per hour, the probability of infection in a susceptible person breathing at rate *p* m^3^ per hour is a function of time (*t* hours):

1 $$ \mathrm{P}\left(\mathrm{t}\right) = 1-exp\left(-\mathrm{q}\mathrm{p}\mathrm{t}/\mathrm{Q}\right) $$

where Q is the volumetric airflow rate in the room (m^3^ per hour). As shown in Equation 1, the traditional Wells-Riley model has not included physical processes that remove infectious agents from air – e.g., loss of infectivity and gravitational settling, but the equation can be modified to address these factors [19]. The model need not be limited to steady-state conditions [20]. A greater limitation of the Wells-Riley equation is exclusive consideration of inhalation exposures. Respiratory infections, however, depending upon the microbe, may arise via multiple routes, motivating the need for dose-response functions.

The U.S. Centers for Disease Control and Prevention (CDC) defines three routes of disease transmission: airborne, contact, and droplet [21]. Airborne transmission involves infectious microbes in small particles that remain infectious over time, allowing dispersal of infectious microbes through the air over long distances. Contact transmission involves the transfer of infectious microbes from one infected person to another, though the transmission may be indirect, via a contaminated object. Droplet is a form of direct contact transmission involving the transfer of infectious microbes in droplets (e.g., relatively large particles) projected from the respiratory tract to the mucosal surfaces of a susceptible person, generally over short distances. These definitions do not represent the full spectrum of pathways by which diseases are transferred. An obvious gap is that droplet-generating activities also produce small particles that can be inhaled in proximity to the infectious person, not just at a long distance. Brosseau and Jones have suggested that aerosol transmission replace, or at least bridge, the definitions of droplet and airborne transmission [22]. In addition, the route of disease transmission refers to the primary route(s) for an infectious agent, though selected settings – e.g., laboratories or healthcare settings – may create opportunities for infection through atypical routes [23]. Accurate representations of the transmission routes are necessary for QMRA, as they drive the assessment of exposure and selection of dose-response function.

The objective of this study is to identify dose-response functions for selected infectious agents capable of causing respiratory infections: *Bordatella pertussis*, rhinovirus, respiratory syncytial virus, and group A *Streptococcus*. To our knowledge, dose-response functions for these microbes have not been published to date, though relevant data exist. These four microbes persist as threats to public health, such that identification of dose-response functions could facilitate exploration of disease transmission and interventions through risk assessment.

## Methods

### Data selection

Studies were compiled over several years as a result of QMRA research activities related to these and other pathogens, not through a systematic review. Studies were identified through searches of the PubMed and Web of Science databases and by cross-referencing cited references. The databases were queried again prior to drafting this article.

One author (RMJ) reviewed all studies to assess whether studies met the data conditions for dose-response analysis. Data met the following conditions for dose-response analysis: *i*) at least three dose levels were used, and *ii*) the number doses with response rate other than 0% or 100% was equal to or greater than the number of dose-response model parameters. These conditions are consistent with recommendations by Haas et al. [12]. Qualitatively, data were also evaluated for relevance for human exposures, with consideration for the route of exposure and outcome studied.

### The infectious microbes

*Bordetella pertussis* is a bacterium that causes the disease pertussis, commonly known as whooping cough. *B. pertussis* cells adhere to cilia of epithelial cells in the upper respiratory tract, initiating symptoms of infection [24,25]. Transmission is generally recognized through the droplet and contact routes [21], but recent animal models suggest that transmission may also occur through the airborne route [26]. As a result, infectivity studies involving aerosol inhalation or deposition into the respiratory tract were judged relevant to disease transmission among humans.

Group A *Streptoccocus* (GAS) include a number of species, the most common being *Streptococcus pyogenes*. GAS infections may involve the upper respiratory tract (pharyngitis) or the skin (impetigo), and invasive disease can develop [27,28]. Here respiratory infection is considered, associated with droplet transmission [21].

Rhinoviruses are a cause of the common cold. Infection occurs primarily in the upper respiratory tract, though the high temperature in the lower respiratory tract does not fully prevent rhinovirus replication [29]. Studies among human volunteers have found transmission to occur when exposed susceptible volunteers do and do not touch their face [30,31]. These data suggest transmission may occur through contact and/or airborne routes, though only droplet transmission is recognized by the CDC [21]. Given these infection sites, experimental exposure to rhinovirus through aerosol and intranasal instillation were judged relevant to natural human infection.

Respiratory syncytial virus (RSV) is a cause of the common cold. In the cotton rat model, virus replication was observed in the alveoli and bronchioles subsequent to intranasal inoculation with RSV [32]. Transmission studies among human volunteers confirmed infection resulting from direct close contact with RSV-infected infants and contact with the eyes and nose after handling RSV-contaminated objects [33]. While the related study of inhalation exposure did not show transmission [33], the result does not preclude transmission through the airborne route due to the potential for low exposure. Virus replication in the alveoli and bronchioles of the cotton rat [32] suggests that inhalation and deposition of RSV in these regions could produce infection. The CDC, however, recognizes only the contract transmission route for RSV [21].

### Dose-response models

Two mechanistic dose-response models were considered: the exponential and beta-Poisson models [12]. The exponential model is a one-hit model, where the probability of infection (or other outcome) given mean dose, d, is expressed

2 $$ {P}_I(d)=1-exp\left(-dk\right) $$

and κ (0 < κ ≤ 1) is the independent and identical probability that an infectious agent survives in the host and initiates infection. The beta-Poisson model is related to the exponential model, but the probability that an infectious agent survives in the host and initiates infection follows a beta distribution. The beta-Poisson model is expressed

3 $$ {P}_I(d)=1-{\left[1+\frac{d}{\beta}\right]}^{-\alpha } $$

where, d is the mean dose, and α and β are parameters of the beta distribution [34]. The median infectious dose, N_50_, is estimated from α and β as:

4 $$ {N}_{50}=\frac{\beta }{2^{1/\alpha }-1} $$

### Dose-response model fit and evaluation

All analyses were implemented using in-house code written by both authors for the R Project for Statistical Computing. Code is available upon request.

Dose-response models were fitted to data using the method of maximum likelihood. Given an experiment with *i =* 1, 2, …, D dose levels, *n*_*i*_ subjects are given mean dose *d*_i_ and *x*_*i*_ subjects (0 ≤ *x*_*i*_ ≤ *n*_*i*_) exhibit the response. The maximum likelihood estimate of the proportion of responding subjects is ${\widehat{p}}_i=\frac{x_i}{n_i}$. The likelihood function is

5 $$ Lik\left(\theta \right)={\prod}_{i=1}^D\left({}_{x_i}^{n_i}\right){p}_i^{x_i}{\left(1-{p}_i\right)}^{n_i-{x}_i} $$

where θ is the dose-response model parameter, and *p*_*i*_ is the probability of response at dose *d*_*i*_ based on the model of choice [12]. For the exponential model, for example, θ = κ and *p*_*i*_ = 1 − exp(−*d*_*i*_*k*). The most-likely value of θ maximizes the likelihood function, and minimizes the negative of the log-likelihood function. The latter approach was implemented using the *optim* function.

Confidence intervals were generated by bootstrapping: Model parameters, θ, were estimated by the method of maximum likelihood to a population randomly sampled with replacement from the study population [12,35]. Given B = 5,000 bootstrapped populations, θ was estimated B times, $\overrightarrow{\theta}=\left\{{\widehat{\theta}}_1,{\widehat{\theta}}_2,\dots, {\widehat{\theta}}_{5,000}\right\}$. For the exponential models, the 95% confidence interval (CI) for the parameter κ is simply the 2.5^th^ and 97.5^th^ percentile of the ordered parameter estimates $\overrightarrow{\theta}$. For the beta-Poisson models, the parameters (α, β) or (α, N_50_) are jointly distributed [12]. The joint distribution of the $\overrightarrow{\theta}$ can be represented graphically. The 95%CI in the response was determined by calculating the response at each dose using each of the B values of $\overrightarrow{\theta}$, and equating the 95%CI with the 2.5^th^ and 97.5^th^ percentile of the ordered response values. The 95%CI for the response is displayed graphically.

Small sample sizes prevented consistent application of Pearson’s χ^2^-test to evaluate model fit. Instead, model fit was evaluated with a permutation test with 100,000 permutations of the Boolean responses [4,36]. The p-value is the proportion of permutations that yield a log-likelihood larger than that observed with the original data. A large p-value (*p* > 0.05) indicates that the exposure has no effect on the outcome because shuffling the exposures yields log-likelihoods as large or larger as the observed data. Comparison between models was based on Akaike’s Information Criterion, AIC = 2k – 2ln(L), where k is the number of parameters in the dose-response model, and ln(L) is the log-likelihood [37,38]. The AIC penalizes models with more parameters.

## Results

### *Bordetella pertussis*

Owing to an interest in vaccine development, the infectivity of *B. pertussis* has been evaluated in many animal models, but only three studies in the mouse model met the data selection criteria. The data are presented in Additional file 1: Table S1.

Halperin et al. [39] exposed 10-day old BALB/C mice to *B. pertussis* strain 18323 through aerosol inoculation and intranasal instillation. In the aerosol study, the unit of dose was the concentration of *B. pertussis* cells per mL of inoculum aerosolized into the exposure chamber. Unfortunately, the concentration of cells in liquid cannot be translated into an airborne concentration or number of inhaled cells, the units of dose relevant to human exposure in the context of disease transmission. As a result, dose-response functions were not fitted to the aerosol study by Halperin et al. [39]. The dose via intranasal instillation was the number of CFU deposited into the nares. Outcomes documented included mortality and positive lung culture upon autopsy: The two outcomes were combined in this analysis to yield a single outcome, infection.

Pittman et al. [40] deposited *B. pertussis* strain 18323 into the external nares of anesthetized mice (Ham/ICR and Anglia strains) aged four weeks or 6-7 days. The outcome was death, occurring > 2 hours and < 24 or 30 days after inoculation. Experiments with mice aged four weeks were pooled because they used the same methods.

Sato et al. [41] exposed mice (DDY and ICR strains) to aerosols of *B. pertussis* Tohama phase I strain during 30 minutes of aerosol generation and 20 minutes of chamber evacuation. Dose was reported as the concentration of *B. pertussis* in the inoculum aerosolized, but in this analysis the dose used was the number of viable cells in the lungs after aerosol exposure, which was determined by autopsy 20 minutes subsequent to exposure. The number of viable cells in the lungs is more directly comparable to human exposure: the fraction of airborne cells that are inhaled and deposit in the lung differ among animals and humans owing to anatomy, respiration rate, animal orientation, etc., but the deposited dose measured in animals can be compared to estimated deposition in the human respiratory tract [42].

For the intranasal studies, AIC recommends the beta-Poisson over the exponential functions (Table 1), which is consistent with graphical display of the models (Additional file 1: Figures S1-S3). In the aerosol study by Sato et al. [41], the two models are functionally equivalent (Additional file 1: Figure S4), and AIC recommends the exponential model because it has only one, rather than two, parameters.

**Table 1:** **Fitted dose-response models for selected agents**

				**Exponential Model**			**Beta-Poisson Model**		
**Host**	**Exposure (unit)**	**Outcome**	**Ref**	κ **(95%CI)**	**AIC** ^**a**^	**p-value** ^**b**^	α**,** β **(N**_**50**_ **)**	**AIC** ^**a**^	**p-value** ^**b**^
*Bordetella pertussis*									
Mouse (10 day old)	Intranasal (CFU)	Infection	[39]	1.14 × 10^−8^	607	0	0.086, 2000	50.9	0.431
				(6.47 × 10^−9^, 3.73 × 10^−8^)			(0.632)		
Mouse (4 week old)	Intranasal (CFU)	Mortality	[40]	6.28 × 10^−8^	58.9	0	0.239, 8.91 × 10^5^	47.3	0.972
				(4.85 × 10^−8^, 8.75 × 10^−8^)			(51900)		
Mouse (6 day old)	Intranasal (CFU)	Mortality	[40]	3.83 × 10^−5^	158	0	0.556, 252	24.2	0
				(1.52 × 10^−5^, 1.04 × 10^−3^)			(102)		
Mouse (21 day old)	Aerosol (CFU inhaled)	Mortality	[41]	2.15 × 10^−6^	8.25	0	3.34, 1.19 × 10^6^	10.0	0
				(1.10 × 10^−6^, 5.76 × 10^−6^)			(5.16 × 10^6^)		
Group A *Streptococcus*									
Mouse	Intranasal (CFU)	Infection	[46]	1.05 × 10^−6^	72.8	0.088	0.341, 1260	15.6	0.308
				(3.19 × 10^−7^, 2.04 × 10^−4^)			(5460)		
Mouse	Intranasal (CFU)	Mortality	[46]	1.38 × 10^−6^	9.22	0	0.762, 1.98 × 10^5^	10.1	<0.001
				(4.59 × 10^−7^, 7.44 × 10^−6^)			(1.34 × 10^5^)		
*Rhinovirus*									
Human	Intranasal (TCID_50_)	Infection	[51]	1.00	11.4	0	0.725, 0.173	12.8	0
				(0.484, 1.00)			(0.108)		
Human (antibody-free)	Intranasal (TCID_50_)	Infection	[50]	0.366	75.2	0.009	0.701, 0.111	16.9	0.427
				(0.125, 1.00)			(0.066)		
Human (antibody-free, pooled)	Intranasal (TCID_50_)	Infection	[50,51]	0.428	89.9	0	0.697, 0.121	25.8	0
				(0.154, 1.00)			(0.071)		
Human (antibody ≤ 4)	Intranasal (TCID_50_)	Infection	[50]	0.138	123	0	0.374, 0.076	24.0	0.358
				(0.074, 0.420)			(0.014)		
*Respiratory Syncytial Virus*									
Human	Intranasal (TCID_50_)	Infection	[55]	9.98 × 10^−5^	5.92	0.002	1.14, 6610	7.69	0.034
				(2.41 × 10^−5^, 5.08 × 10^−4^)			(7900)		
Human (pooled)	Intranasal (TCID_50_)	Infection	[54,55]	2.34 × 10^−5^	20.3	0.014	0.217, 1380	16.0	0.080
				(1.18 × 10^−5^, 4.42 × 10^−5^)			(59.0)		
Human (pooled)	Intranasal (TCID_50_)	Infection	[53-55]	2.86 × 10^−5^	95.5	0.572	0.026, 8.59 × 10^−8^	39.6	0.999
				(1.65 × 10^−5^, 5.60 × 10^−5^)			(2.27 × 10^−19^)		

^a^AIC is Akaike’s Information Criterion.

^b^p-value is for the permutation test.

Comparing the two intranasal dose-response studies with mortality outcome, *B. pertussis* appears to be more infectious among younger mice. In the exponential functions, this is indicated by larger values of κ (Table 1). In the better-fit beta-Poisson functions, infectivity is most readily compared by the N_50_, which equals 102 CFU and 51,900 CFU for the 6 day old and 4 week old mice, respectively. As expected, the probability of infection is lower than the probability of mortality: For the infection outcome, N_50_ = 0.632 CFU. *B. pertussis* appears substantially less infectious through aerosol exposure, for which N_50_ = 5.16 × 10^6^ CFU. Differences in infectivity with age have been observed for other organisms, like influenza, in animal models and epidemics [43].

### Group A *streptococci* (GAS)

Owing to the focus on respiratory infection, human dose-response studies using intradermal injection [44] and wound inoculation [45] were not evaluated. Exposure though the respiratory tract has only been studied among mice, by Wessels and Bronze [46], who administered GAS (M type 24 strain Vaughn) via intranasal instillation. The study outcomes were mortality and infection, where infection was defined by pharyngeal swab culture positive for GAS (Additional file 1: Table S2). Experiments involving the mutant strain were not used.

For both outcomes, AIC recommends the beta-Poisson model (Table 1). Confidence intervals are narrower for the mortality-based dose-response models than for the infection-based models (Additional file 1: Figures S5 and S6). As indicated by N_50_, higher doses are required to obtain 50% mortality than 50% infectivity (Table 1).

### Rhinovirus

Aerosol infectivity studies of human volunteers have little information for dose-response modeling because they observed 100% of volunteers to become infected: Exposures were ≥ 16 TCID_50_ [47] and ≥ 17 TCID_50_ rhinovirus NIH 1734 [48]. Two of three intranasal infectivity studies identified met the inclusion criteria and were evaluated (Additional file 1: Table S3) [49-51].

Hendley et al. [50] inoculated volunteers with varied levels of serum antibodies intranasally with rhinovirus type 39. Higher infectivity was observed among volunteers with lower antibody levels prior to inoculation (Tables 1 and S3), which makes sense because higher antibody levels are indicative of immunity. Regardless of pre-inoculation antibody level (antibody-free or ≤4), AIC recommends the beta-Poisson dose-response models (Table 1), which qualitatively provide a better representation of the data than the exponential model (Additional file 1: Figures S7 and S8).

In contrast, AIC recommends the exponential model for intranasal infectivity of rhinovirus type 16 observed by D’Alessio et al. [51]. The maximum likelihood estimate of the exponential model parameter is κ = 1, indicating that each rhinovirus can initiate infection. As κ must be less than or equal to one, the upper 95% confidence interval coincides with the maximum likelihood estimate (Additional file 1: Figure S9).

The two studies used different strains of rhinovirus – types 16 and 39, and the values of κ and N_50_ suggest that type 16 may be more infectious than type 39 (Table 1). When data for the two rhinoviruses among antibody-free volunteers were pooled, the fitted dose-response functions had κ and N_50_ values between those observed for each rhinovirus type (Table 1, Additional file 1: Figure S10).

### Respiratory syncytial virus

While numerous animal models have been used to study RSV infection [52], none met the data selection criteria. Of the studies with human volunteers [53-56], only one study met the data selection criteria: Hall et al. [55] inoculated human volunteers with RSV strain A2 in through the nose or eyes (Additional file 1: Table S4). Two other studies [53,54] were considered for pooling with Hall et al. because they used RSV strain A2 and a similar study design (Additional file 1: Table S4).

For the Hall et al. [55] data alone, the two dose-response models are functionally equivalent (Additional file 1: Figure S11), and AIC recommends the exponential model (Table 1). However, when pooled with Lee et al. [54], the beta-Poisson model is recommended by AIC (Table 1, Additional file 1: Figure S12). The addition of observations by Mills et al. [53] offered no improvement, as indicated by the increased p-value and AIC (Table 1, Additional file 1: Figure S13).

## Discussion

In disease transmission, the critical step is that an infectious microbe moves through the environment to a site in a susceptible person where it is capable of initiating infection. To accurately quantify risk of infection, a dose-response function should be based on experimental data that represent this process. In studies with human volunteers, this means the exposure conditions and doses administered should reflect natural transmission conditions. In studies with animal models, there are additional host-specific concerns. In ideal situations, the infection sites are in similar anatomical locations in the animal model and humans, and the fraction of airborne microbes that reach the infection sites is the same, or can be corrected for based on geometry of the respiratory tract, animal orientation, respiratory rate and other factors. Often, the conditions are not ideal, and important uncertainties are introduced to the extrapolation of an animal-based dose-response function to humans.

Intranasal instillation is an exposure route commonly used in animal models and human subjects, but it is difficult to interpret for natural disease transmission. Intranasal instillation involves the introduction of a small volume of fluid containing infectious microbes into the nares: Given sufficient fluid volume, instilled materials have been shown to move to the lungs [57,58]. The disease transmission routes that best align with exposures through intranasal instillation are unclear. In contact transmission, infectious microbes likely deposit on the exterior of the nares and/or on the mucous membranes just inside the nares, surfaces readily accessible to the hands. In droplet transmission, particles containing infectious microbes may project onto or into the nares. In airborne transmission, inhaled particles may deposit throughout the respiratory tract, including the head airways [42]. While the deposition patterns of inhaled particles in the human respiratory tract is well understood [42], the dispersion of the infectious microbes in the head airways subsequent to contact and droplet exposure is unknown. Thus, it is particularly difficult to equate the dose administered through intranasal instillation with exposures through the contact and droplet routes.

Mice were the animal models used in experimental infectivity studies for *B. pertussis* and GAS (Table 1); and are one of many animal models used to study the infectivity and pathogenesis of infectious microbes, the toxicity of chemicals, and disease. The selection of an animal model considers a variety of factors, including: similarity of the respiratory tract, microbe tropism, immune system and response, and symptom presentation between the animal model and humans (see for example the discussion of guinea pigs and ferrets for influenza [59,60]). The presentation of symptoms in the animal model is very important in studies of disease transmission among animals since the route of transmission should be as similar to that in humans as possible; but is likely less important in evaluating a dose-response function. Unfortunately, unless studies have been performed in both humans and animal models it is not possible to evaluate the equivalency.

The objective of this study was to fit dose-response models for selected microbes capable of infecting the respiratory tract, so as to facilitate QMRA. Multiple functions were identified for *B. pertussis*, GAS, rhinovirus, and RSV (Table 1). Though statistical criteria, such as AIC, indicate the relative performance of two dose-response functions fitted to a specific data set (exponential or beta-Poisson), the selection of a dose-response function from among those fitted to multiple data sets is a context-specific decision that depends upon the specific question to be addressed through the risk assessment. There are many dimensions in the interpretation of dose-response functions fitted to data from animal models and human volunteers for natural disease transmission that are more important than questions about the study design (e.g., number of subjects, etc.) and statistical measures of model fit. These dimensions include, but are not limited: tropism of the microbe for the host species, anatomical location of sites susceptible to infection, and the unit of exposure and its relation to the number of infectious microbes that reach sites susceptible to infection.

Owing to the importance of the risk assessment context, the fact that few of the dose-response functions yielded an objectively good representation of the data (see Figures in Additional file 1), differences between strains, and uncertainty about extrapolation of animal-based dose-response functions to human disease transmission, we have declined to recommend a single dose-response function for each of the infectious microbes in this study (Table 1). Future work to understand infection processes in more detail for these microbes is necessary, not only for the sake of general knowledge, but also because the diversity of plausible dose-response functions for each microbe may yield different estimates of infection risk, and different risk management conclusions. Two key data gaps are the fate of microbes deposited onto the facial mucous membranes by contact and droplet spray, and the relative distribution of infection sites in the respiratory tract of animal hosts and humans. Work with influenza virus demonstrates the feasibility of the later studies (see [61]).

Frequently, the beta-Poisson model was preferred over the exponential model (Table 1). This is reassuring because the beta-Poisson model allows for biologically-plausibility inter-individual variability in host susceptibility and virus infectivity. Exponential models were preferred when few subjects and few unique doses were tested: In these conditions, there is insufficient data to characterize inter-individual variability and a model with fewer parameters is preferred by AIC.

Despite the limitations identified for the fitted dose-response functions (Table 1), the availability of these functions makes it possible to apply QMRA to explore questions of significance to public health decision-making. For example, QMRA can be used in conjunction with exposure models and epidemiologic models to explore the effectiveness of infection control interventions in healthcare settings, including personal protective equipment (PPE) and work practices, like patient isolation and hand hygiene. PPE are specialized clothing or equipment worn by workers or other susceptible people to prevent exposure, including: gloves, gowns, eye protection, respirators, facemasks, etc. Infection control interventions are recommended based on observational studies that indicate association of the intervention(s) with lower risk of infection [21], and while the effectiveness of most interventions are biologically plausible, quantitative measures of effectiveness against specific infectious microbes and contract transmission routes that would help to prioritize interventions are often not available [62]. The limitations of the dose-response functions can be addressed in QMRA through uncertainty analysis, and/or by focusing on the change in risk resulting from interventions rather than the absolute value of risk.

QMRA is an important tool for understanding and mitigating infection risk. While limitations of dose-response functions have been described for these agents, there are also significant limitations and uncertainties about other aspects of QMRA, including exposure assessment. Quantitative measurements of infectious microbe emission and exposure are extremely limited, so the parameterization and verification of exposure models are difficult. Nonetheless, QMRA provides a transparent, systematic process by which to understand and evaluate risks posed by infectious microbes. With the new dose-response functions presented herein, the application of QMRA to pertussis, GAS pharyngitis, and viruses causing the common cold can begin.

## Conclusions

Dose-response functions were fitted for *B. pertussis*, GAS, rhinovirus, and RSV; though all functions have limitations associated with the use of an animal model, intranasal instillation, susceptibility of the hosts, etc. The persistence of these microbes as threats to public health, however, motivate application of QMRA to understand the magnitude of infection risks and identify effective controls to reduce risk. Using the dose-response functions fitted in this study, QMRA can begin to be applied.

## Additional file

Additional file 1: **Supplementary materials.** The supplementary materials contains the dose-response data analyzed and graphical prescutations of model fit.

## Abbreviations

AIC: Akaike’s Information Criterion
CDC: U.S. Centers for Disease Control and Prevention
GAS: Group A *Streptococcus*
PPE: Personal Protective Equipment
QMRA: Quantitative microbial risk assessment
RSV: Respiratory Syncytial Virus
