# Supplementary material for: Dose-response models for selected respiratory infectious agents: Bordetella pertussis, group a Streptococcus, rhinovirus and respiratory syncytial virus
Source: BMC Infect Dis. 2015 Feb 24;15:90. doi: 10.1186/s12879-015-0832-0 (PMC4345006; doi:10.1186/s12879-015-0832-0)
Supplement: Supplementary file 1 — Supplementary materials. The supplementary materials contains the dose-response data analyzed and graphical prescutations of model fit. [file 12879_2015_832_MOESM1_ESM.docx]

Dose-Response Models for Selected Respiratory Infectious Agents: *Bordetella pertussis*, group A *Streptococcus*, rhinovirus and respiratory syncytial virus

Rachael M. Jones*

Yu-Min Su

Additional file

Table S1. Dose-response data for *Bordetella pertussis* among mice*.* Halperin et al. [1] observed infection (mortality or positive lung culture) subsequent to intranasal instillation. Pittman et al. [2] observed mortality subsequent to intranasal instillation. Sato et al. [3] observed mortality subsequent to aerosol exposure, where dose is the number of viable cells in the lungs.

|  |  | **Number of Mice** | | **Percent**  **With Outcome** |
| --- | --- | --- | --- | --- |
|  | **Dose (CFU)** | **With Outcome** | **Exposed** |  |
| **Halperin et al.** | | |  |  |
|  | 1.4 × 10^2^ | 0 | 28 | 0 |
|  | 1.4 × 10^3^ | 2 | 28 | 7 |
|  | 1.9 × 10^4^ | 13 | 78 | 17 |
|  | 3.3 × 10^4^ | 7 | 20 | 35 |
|  | 2.5 × 10^5^ | 7 | 40 | 18 |
|  | 1.0 × 10^6^ | 7 | 15 | 47 |
|  | 5.0 × 10^6^ | 15 | 22 | 68 |
|  | 1.0 × 10^7^ | 6 | 15 | 40 |
|  | 4.0 × 10^7^ | 15 | 22 | 68 |
|  | 2.0 × 10^8^ | 11 | 20 | 55 |
|  | 3.0 × 10^9^ | 6 | 8 | 75 |
| **Pittman et al. (aged 4 weeks)** | | | |  |
|  | 1.37 × 10^5^ | 1 | 50 | 2 |
|  | 2.2 × 10^5^ | 1 | 15 | 7 |
|  | 3.0 × 10^5^ | 6 | 44 | 14 |
|  | 1.35 × 10^6^ | 5 | 50 | 10 |
|  | 2.2 × 10^6^ | 6 | 14 | 43 |
|  | 1.35 × 10^7^ | 18 | 50 | 36 |
|  | 2.2 × 10^7^ | 14 | 15 | 93 |
| **Pittman et al. (aged 6-7 days)** | | | |  |
|  | 0.7 × 10^1^ | 1 | 15 | 7 |
|  | 0.7 × 10^2^ | 0 | 14 | 0 |
|  | 0.7 × 10^3^ | 9 | 17 | 53 |
|  | 0.7 × 10^4^ | 19 | 20 | 95 |
|  | 0.7 × 10^5^ | 18 | 19 | 95 |
|  | 0.7 × 10^6^ | 17 | 18 | 94 |
|  | 0.7 × 10^7^ | 19 | 19 | 100 |
| **Sato et al.** | | | |  |
|  | 2.3 × 10^4^ | 10 | 0 | 0 |
|  | 1.0 × 10^5^ | 10 | 30 | 30 |
|  | 1.2 × 10^6^ | 10 | 90 | 90 |

Figure S1. Dose-response models fitted to the infection outcome observed by Halperin et al. [1] in mice exposed intranasally to *B. pertussis*.

Figure S2. Dose-response models fitted to the mortality outcome observed by Pittman et al. [2] among 6-7 day old mice exposed intranasally to *B. pertussis*.

Figure S3. Dose-response models fitted to the mortality outcome observed by Pittman et al. [2] among 4 week old mice exposed intranasally to *B. pertussis*.

Figure S4. Dose-response models fitted to the mortality outcome observed by Sato et al. [4] among mice exposed to *B. pertussis* aerosols.

Table S2. Pharyngeal colonization (positive throat culture) and death in mice intranasally exposed to GAS by Wessels and Bronze. [5]

| **Inoculum**  **(CFU)** | **Number**  **Exposed** | **Pharyngeal**  **Culture Positive** | | **Mortality** | |
| --- | --- | --- | --- | --- | --- |
|  |  | **Number** | **Percent** | **Number** | **Percent** |
| 10^3^ | 5 | 0 | 0 | 0 | 0 |
| 10^4^ | 5 | 4 | 80 | 0 | 0 |
| 10^5^ | 5 | 4 | 80 | 2 | 40 |
| 10^6^ | 5 | 5 | 100 | 3 | 60 |
| 10^7^ | 5 | 4 | 80 | 5 | 100 |

Figure S5. Dose-response models fitted to the infection outcome (positive pharyngeal culture) observed by Wessels and Bronze [5] among mice subsequent to intranasal instillation of GAS.

Figure S6. Dose-response models fitted to the mortality outcome observed by Wessels and Bronze [5] among mice subsequent to intranasal instillation of GAS.

Table S3. Rhinovirus type 39 infection observed by Hendley et al. [6] and rhinovirus type 16 infection observed by D’Alessio et al. [7] among human volunteers inoculated by intranasal instillation.

|  | **Dose**  **(TCID_50_)** | **Number** | | **Percent**  **Infected** |
| --- | --- | --- | --- | --- |
|  |  | **Exposed** | **Infected** |  |
| **Hendley et al.** [6] **Antibody-Free Volunteers** | | | | |
|  | 0.05 | 2 | 0 | 0 |
|  | 0.15 | 4 | 1 | 25 |
|  | 0.5 | 7 | 5 | 71 |
|  | 1.5 | 19 | 18 | 95 |
|  | 5 | 1 | 1 | 100 |
|  | 50 | 20 | 19 | 95 |
| **Hendley et al.** [6] **Low-Antibody Volunteers** | | | | |
|  | 0.05 | 6 | 0 | 0 |
|  | 0.15 | 6 | 2 | 33 |
|  | 0.5 | 13 | 7 | 54 |
|  | 1.5 | 26 | 21 | 81 |
|  | 5 | 8 | 5 | 63 |
|  | 50 | 37 | 33 | 89 |
| **D’Alessio et al.** [7] | | | | |
|  | 0.01 | 5 | 1 | 20 |
|  | 0.1 | 2 | 0 | 0 |
|  | 1.1 | 5 | 3 | 60 |
|  | 2.2 | 3 | 3 | 100 |
|  | 110 | 5 | 5 | 100 |
|  | 1,100 | 15 | 15 | 100 |
|  | 11,000 | 3 | 3 | 100 |

Figure S7. Dose-response models fitted to the infection outcome observed by Hendley et al. [6] among antibody-free human volunteers subsequent to intranasal instillation of rhinovirus type 39.

Figure S8. Dose-response models fitted to the infection outcome observed by Hendley et al. [6] among human volunteers with low (≤ 4) serum antibody subsequent to intranasal instillation of rhinovirus type 39.

Figure S9. Dose-response models fitted to the infection outcome observed by D’Alessio et al. [7] among human volunteers inoculated intranasally with rhinovirus type 16.

Figure S10. Dose-response models fitted to the infection outcome observed by Hendley et al. [6] among antibody-free human volunteers subsequent to intranasal instillation of rhinovirus type 39 pooled with type 16 infection observed by D’Alessio et al. [7].

Table S4. Infection by respiratory syncytial virus A2, indicated by viral shedding, among human volunteers subsequent to intranasal instillation. Hall et al. [8] observed identical outcomes for volunteers inoculated in the nose and eye. Data shown for Mills et al. [9] are only for subjects with low pre-exposure serum antibodies.

|  |  | **Number of Volunteers** | | **Percent**  **Infected** |
| --- | --- | --- | --- | --- |
|  | **Dose (CFU)** | **Infected** | **Exposed** |  |
| **Hall et al.**  [8] | | |  |  |
|  | 1.6 × 10^2^ | 4 | 0 | 0 |
|  | 1.6 × 10^3^ | 4 | 1 | 25 |
|  | 1.6 × 10^5^ | 4 | 3 | 75 |
| **Lee et al.** [10] | | | |  |
|  | 5.0 × 10^3^ | 14 | 3 | 21 |
|  | 5.0 × 10^4^ | 14 | 7 | 50 |
| **Mills et al.** [9] | | | |  |
|  | 5.0 × 10^2^ | 8 | 8 | 100 |
|  | 1.0 × 10^5^ | 6 | 4 | 67 |

Figure S10. Dose-response models fitted to the infection outcome observed by Hall et al. [8] among human volunteers inoculated through the eyes and nose with RSV strain A2.

Figure S11. Dose-response models fitted to the infection outcome observed by Hall et al. [8] and Lee et al. [10] among human volunteers inoculated through the eyes and nose with RSV strain A2.

Figure S12. Dose-response models fitted to the infection outcome observed by Hall et al., [8] Lee et al. [10] and Mills et al. [9] among human volunteers inoculated through the eyes and nose with RSV strain A2.

References

1. Halperin SA, Heifetz SA, Kasina A: **Experimental respiratory infection with *Bordetella pertussis* in mice: comparison of two methods.** Clin Invest Med 1988, **11**:297-303.

2. Pittman M, Furman BL, Wardlaw AC: ***Bordetella pertussis*respiratory tract infection in the mouse: pathophysiological responses.** J Infect Dis 1980, **142**:56-66.

3. Sato H, Sato Y: ***Bordetella pertussis*infection in mice: correlation of specific antibodies against two antigens, pertussis toxin, and filamentous hemagglutinin with mouse protectivity in an intracerebral or aerosol challenge system.** Infect Immun 1984, **46**:415-421.

4. Sato Y, Izumiya K, Sato H, Cowell JL, Manclark CR: **Aerosol infection of mice with *Bordetella pertussis*.** Infect Immun 1980, **29**:261-266.

5. Wessels MR, Bronze MS: **Critical role of the group A streptococcal capsule in pharyngeal colonization and infection in mice.** Proc Natl Acad Sci USA 1994, **91**:12239-12242.

6. Hendley JO, Edmondson J, W.P., Gwaltney J, J.M.: **Relation between naturally acquired immunity and infectivity of two rhinoviruses in volunteers.** J Infect Dis 1972, **125**:243-248.

7. D'Alessio DJ, Meschievitz CK, Peterson JA, Dick CR, Dick EC: **Short-duration exposure and the transmission of rhinoviral colds.** J Infect Dis 1984, **150**(2):189-194.

8. Hall CB, Douglas J, R.G., Schnabel KC, Geiman JM: **Infectivity of respiratory syncytial virus by various routes of inoculation.** Infect Immun 1981, **33**:779-783.

9. Mills J, Van Kirk JE, Wright PF, Chanock RM: **Experimental respiratory syncytial virus infection of adults.** J Imunol 1971, **107**:123-130.

10. Lee EH, Walsh EE, Falsey AR, Betts RF, Treanor JJ: **Experimental infection of humans with A2 respiratory syncytial virus.** Antiviral Research 2004, **63**:191-196.
